# Aspects cliniques, électrocardiographiques et échocardiographiques de l’hypertendu âgé au Sénégal

**DOI:** 10.11604/pamj.2016.25.77.10086

**Published:** 2016-10-17

**Authors:** Simon Antoine Sarr, Kana Babaka, Mouhamadou Cherif Mboup, Pape Diadie Fall, Khadidiatou Dia, Malick Bodian, Mouhamadou Bamba Ndiaye, Adama Kane, Maboury Diao, Serigne Abdou Ba

**Affiliations:** 1 Service de Cardiologie, Centre Hospitalier Universitaire Aristide Le Dantec, Dakar Etoile, Senegal; 2 Service de Cardiologie, Hôpital Principal de Dakar, Sénégal

**Keywords:** Hypertendus âgés, ECG, échocardiographie Doppler, Elderly hypertensive patients, ECG, Doppler echocardiography

## Abstract

**Introduction:**

L’hypertension artérielle (HTA) du sujet âgé est un facteur indépendant de maladie cardio-vasculaire. Nos objectifs étaient de décrire les aspects cliniques, électrocardiographique et échocardiographiques de l’HTA du sujet âgé.

**Méthodes:**

Nous avons mené une étude descriptive et transversale de Janvier à Septembre 2013. Etaient inclus les sujets hypertendus âgés d’au moins 60 ans suivis en ambulatoire au service de cardiologie de l’Hôpital Principal de Dakar. Les données statistiques étaient analysées par le logiciel Epi Info 7 et une valeur de p < 0,05 était retenue comme significative.

**Résultats:**

Au total, 208 patients étaient inclus. L’âge moyen était de 69,9 ans avec une prédominance féminine (sex-ratio de 0,85). La pression artérielle moyenne était de 162/90mmHg. L’HTA était contrôlée dans 13% des cas. A l’électrocardiogramme, on notait un trouble du rythme (17,78%), une hypertrophie auriculaire gauche (45,19%), une hypertrophie ventriculaire gauche (28,85%) et 2 cas de bloc auriculo-ventriculaire complet. Le Holter ECG révélait 4 cas de tachycardie ventriculaire non soutenue (IVb de Lown), 6 cas de fibrillation atriale paroxystique et 1 cas de flutter atrial paroxystique. L’échocardiographie réalisée chez 140 patients retrouvait une HVG à prédominance concentrique chez 25 patients, plus fréquente chez les hommes (p=0,04) et une dilatation de l’oreillette gauche dans 56,42% des cas, plus fréquente chez les patients plus âgés (p= 0,01).

**Conclusion:**

Les aspects électrocardiographiques et échocardiographiques dans la population hypertendue âgée sont caractérisés par l’hypertrophie ventriculaire gauche notamment concentrique, la fréquence des arythmies révélées quelques fois par l’enregistrement électrocardiographique de longue durée.

## Abstract (en)

**Introduction:**

Arterial hypertension (HTA) in the elderly is an independent risk factor for cardiovascular disease. Our study aims to describe the clinical, electrocardiographic and echocardiographic aspects of Arterial hypertension in elderly patients.

**Methods:**

We conducted a descriptive, cross-sectional study from January to September 2013. Hypertensive patients =60 years treated in Outpatient Cardiology Department at the Principal Hospital in Dakar were included in the study. Statistical data were analyzed using Epi Info 7 software and a p-value < 0.05 was taken as significant.

**Results:**

A total of 208 patients were enrolled in the study. The average age was 69.9 years with a female predominance (sex ratio 0.85). Average blood pressure was 162/90 mm Hg. HTA was under control in 13% of cases. The ECG showed evidence of rhythm disturbance (17.78%), left atrial enlargement (45.19%), left ventricular hypertrophy (28.85%) and complete atrioventricular block in 2 cases. Holter ECG revealed non-sustained ventricular tachycardia (Lown class IVb) in 4 cases, paroxysmal atrial fibrillation in 6 cases and paroxysmal atrial flutter in 1 case. Echocardiography performed in 140 patients showed mainly concentric left ventricular hypertrophy in 25 patients, occuring more frequently in males (p=0,04) and dilated left atrium in 56,42% of cases, occuring more frequently in elderly patients (p= 0,01).

**Conclusion:**

Electrocardiographic and echocardiographic aspects in elderly hypertensive population are characterized by concentric left ventricular hypertrophy and by the frequency of arrhythmias sometimes revealed by long-term continuous external electrocardiographic recording.

## Introduction

L’hypertension artérielle (HTA) du sujet âgé est un facteur indépendant de maladie cardio-vasculaire. De même, il s’agit d’un indicateur indépendant et puissant significativement associé à l’augmentation de la morbi-mortalité par le biais de l’infarctus du myocarde, l’insuffisance cardiaque, l’insuffisance rénale et de l’atteinte cérébro-vasculaire [1]. Sa prévalence augmente avec l’âge. D’après l’étude MONA LISA, elle est en France de 80% et 71%, respectivement chez les hommes et les femmes, dans la classe d’âge 65-74 ans [2]. En Afrique sub-saharienne, la prévalence de l’HTA dans la tranche d’âge de 60-69 ans chez les hommes et chez les femmes atteint des taux respectifs de 57,4% et 61,5% [3]. Le nombre d’hypertendus ne cessera d’augmenter, et atteindra selon l’OMS le nombre de 150 millions en 2025 [3]. Ces prévisions seraient liées entre autres, au vieillissement progressif de la population africaine [3]. Nous avons réalisé ce travail dans le but de déterminer les caractéristiques cliniques, électrocardiographiques et échocardiographiques des hypertendus âgés de plus de 60 ans.

## Méthodes

Nous avons réalisé une étude descriptive, transversale sur une durée de neuf mois, de Janvier à Septembre 2013. Elle incluait les patients hypertendus âgés d’au moins 60 ans suivis en ambulatoire au service de cardiologie de l’Hôpital Principal de Dakar. Il s’agissait de recueillir les signes fonctionnels et les données de l’examen physique; d’enregistrer un électrocardiogramme (ECG) de surface de manière systématique à la recherche d’une hypertrophie ventriculaire gauche (HVG), de troubles du rythme et de la conduction mais aussi de signes d’insuffisance coronarienne. L’échocardiographie était réalisée en cas d’indication, il s’agissait de mesurer la taille des cavités cardiaques, de déterminer la masse ventriculaire gauche (indexée à la surface corporelle), d’étudier les fonctions systolique et diastolique du ventricule gauche. Un Holter ECG était réalisé en cas d’indication, à la recherche de troubles du rythme ou de la conduction paroxystiques. L’HVG était définie à l’ECG par un indice de Sokolow-Lyon (SV1+RV5 ou RV6)>35 mm et/ou un indice de Cornell (SV3+RavL) > 28 mm chez l’homme et > 20 mm chez la femme. A l’échocardiographie, l’HVG était définie par une masse ventriculaire gauche (MVG) indexée > 115g/m^2^ chez les hommes et 95 g/m2 chez la femme [4]. Le profil mitral a également été étudié et classé en 3 types selon les critères de l’American Society of Echocardiography (ASE) [5]. Les données étaient relevées sur une fiche pré-établie et enregistrées sur une base Excel. L’analyse et les tests statistiques étaient réalisés par le logiciel Epi info 7. Il s’agissait d’étudierles variables et leur distribution mais aussi de faire une comparaison de moyenne et de proportion. Une valeur de p<0,05 était définie comme statistiquement significative.

## Résultats

Nous avons inclus 208 patients sur un nombre total de 1080 patients consultés pendant la période d’étude. La prévalence de l’HTA chez le sujet âgé était de 19,26%. L’âge moyen était de 69,9 ± 7,6 ans [60-91 ans]. La tranche d’âge la plus représentée était celle comprise entre 60 et 70 ans (60%). On notait une prédominance féminine avec un sex-ratio de 0,85. L’hypertension systolo-diastolique prédominait (74,4%). Cent soixante-treize (173) patients (83,17%) étaient déjà suivis pour une HTA. La durée moyenne d’évolution de l’HTA était de 9,6 ± 8,26 ans [1 mois - 42 ans]. Concernant les données cliniques, parmi les signes de Dieulafoy, les céphalées prédominaient (25,5%) suivies des acouphènes (17,3%), des vertiges (15,4%) et du flou visuel (7,5%). Par ailleurs, nous avions retrouvé une dyspnée, des palpitations et des douleurs thoraciques respectivement dans 19,7%, 12,5% et 5,3% des cas. La pression artérielle systolique moyenne était de 162 mmHg [100-220 mmHg]; celle diastolique moyenne était de 90 mmHg [70-150 mmHg]. L’HTA était contrôlée chez 13% des patients. Les anomalies cardio-vasculaires sont listées dans le Tableau 1. Quant aux données électrocardiographiques, le rythme sinusal était noté dans 96,8% des cas. Les anomalies retrouvées à l’ECG sont présentées dans le Tableau 2. Un Holter ECG avait été fait chez 42 patients. Les différentes anomalies notées sont regroupées dans le Tableau 3. L’échocardiographie Doppler était effectuée chez 140 patients soit 67,3% de notre population. La MVG moyenne était de 86,33 ±4,27 g/m² [3,5-181,1 g/m^2^]. L’HVG était retrouvée chez 25 patients (17,85%), à prédominance concentrique (92%) contre 8% d’HVG excentrique. Les hommes présentaient significativement plus d’HVG (p=0,04). La surface moyenne de l’oreillette gauche était de 20,7 ± 6,9cm². Une dilatation de l’oreillette gauche était observée chez 56,42% des patients et était significativement plus fréquente chez les patients plus âgés (p= 0,01). Les troubles du rythme étaient significativement plus fréquents chez les patients qui avaient une dilatation de l’OG (p= 0,02). La moyenne de la fraction d’éjection systolique du VG était de 67,09 ± 12,47%. Une dysfonction ventriculaire gauche était notée dans 5,7% et des troubles segmentaires de la contractilité dans 4 cas. Un profil mitral de type I, II et III était respectivement retrouvé dans 88,57%, 9,28% et 2,14 %. Les données retrouvées à l’échocardiographie Doppler sont regroupées dans le Tableau 4.

**Tableau 1:** répartition des anomalies cardio-vasculaires parmi les sujets âgés hypertendus

Anomalies cardio-vasculaires	Fréquence	Pourcentage (%)
Irrégularité du rythme	20	9,8
B4 gauche	13	6,2
**Souffles systoliques**		
Aortique	11	5,2
Mitral	11	5,2
Souffle diastolique aortique	1	0,4

**Tableau 2:** anomalies retrouvées à l’électrocardiogrammedes sujets âgés hypertendus

Anomalies	Fréquence	Pourcentage (%)
Troubles du rythme	**37**	**17,7**
Extrasystoles ventriculaires	18	8,6
Extrasystoles atriales	15	7,2
Flutter atrial	6	2,8
Fibrillation atriale	1	0,4
Hypertrophie atriale gauche	**94**	**45,2**
Hypertrophie ventriculaire gauche (HVG)	**60**	**28,8**
HVG systolique	32	15,3
HVG diastolique	28	13,3
Troubles de la repolarisation	**64**	**30**
Troubles de la conduction	**54**	**25,9**
Hémibloc antérieur isolé	30	14,4
Bloc de branche gauche complet	4	1,9

Troubles de la repolarisation : ischémie sous épicardique, nécrose à l’électrocardiogramme

**Tableau 3:** anomalies notées au Holter ECG des sujets âgés hypertendus

Anomalies	Fréquence
Hyperexcitabilité ventriculaire	**18**
Classe II de Lown	6
Classe III de Lown	4
Classe IVa de Lown	4
Classe IVb de Lown	4
Hyperexcitabilité atriale	**15**
Fibrillation atriale paroxystique	6
Flutter atrial paroxystique	1
Extrasystoles atriales multiples polymorphes	8
Episodes de bradycardie importante (FC<40/min)	**2**

**Tableau 4:** anomalies échocardiographiques retrouvées chez les sujets âgés hypertendus

Résultats échographiques		Fréquence (n=140)	Pourcentage (%)
Hypertrophie ventriculaire gauche (HVG)		25	17,8
Dilatation de l’oreillette gauche		79	56,4
Troubles segmentaires de la contractilité du ventricule gauche		4	2,8
Dysfonction ventriculaire gauche		8	5,7
Dysfonction diastolique	Type 1	124	88,5
	Type 2	13	9,2
	Type 3	3	2,1
Bourrelet septal sous-aortique non obstructif		45	32,1
Calcification de la valve aortique		18	12,8
Hypertension artérielle pulmonaire (HTAP)		7	5
Anévrysme du septum inter-atrial		6	4,2
Calcifications des deux valves (aortique et mitrale)		4	2,8
Rétrécissement aortique serré		3	2,1
Dilatation de l’oreillette droite		2	1,4
Calcification isolée de la valve mitrale		1	0,7
Séquelles rhumatismales aortique et mitrale		1	0,7

## Discussion

Les hypertendus âgés occupent une part significative de l’ensemble des patients suivis en ambulatoire dans notre pratique. Ceci s’explique d’une part par la prévalence de l’HTA dans la population Sénégalaise: 25 à 30% [6], mais aussi par l’incidence plus grande de l’HTA chez le sujet âgé pouvant atteindre 60 à 80% [7]. L’examen clinique est une étape importante de l’évaluation de l’hypertendu. Il s’agit essentiellement de rechercher une HTA secondaire, une complication ou des facteurs de risque cardio-vasculaire associés [4]. L’électrocardiogramme est un examen simple, accessible, peu coûteux permettant de détecter des complications de l’HTA. Par ailleurs, il peut fournir des éléments pronostiques. L’échocardiographie est beaucoup plus précise pour la détection et la quantification de l’HVG. Elle permet une meilleure approche du risque cardio-vasculaire global et permet quelques fois de guider le traitement [4]. Dans notre travail, nous avions retrouvé 28,85% de cas d’HVG électrique et 17,85% d’HVG échographique à prédominance concentrique. Ces données sont inférieures à celles retrouvées dans l’étude MONALISA (HVG échographique 41%) [2] et à la prévalence de 53,3% d’HVG par Niakara chez le Noir Africain hypertendu [8]. Dans une population âgée en moyenne de 55 ans, Jaleta retrouvait en Ethiopie une prévalence de l’HVG de 52% [9]. L’HVG chez l’hypertendu, diagnostiquée sur des critères électrocardiographiques [10] et échocardiographiques, est un facteur prédictif majeur d’augmentation de l’incidence de maladies coronaires, d’infarctus du myocarde, d’accident vasculaire cérébral, d’insuffisance cardiaque, d’arythmies ventriculaires et de mort subite [11], indépendamment du niveau de pression artérielle et des autres FDR [12]. Monfared, dans son travail, faisait le lien étroit de l’HVG et de la microalbuminurie dans une population d’hypertendus [13].

Nous n’avons pas retrouvé de relation statistiquement significative entre l’HVG et ces paramètres. C’est plutôt le sexe qui parait déterminant, ceci malgré une prise en compte du genre quant à la définition de l’HVG. Les troubles du rythme, sur l’ECG de base, étaient retrouvés dans 17,78% des cas. Ils étaient dominés par les extrasystoles en général, et celles ventriculaires en particulier. Nous n’avions pas trouvé de relation statistiquement significative entre l’HVG et les troubles du rythme cardiaque. Pose-Reino faisait le même constat dans son travail comparant deux groupes de patients hypertendus et non hypertendus [14]. Seule la dilatation de l’oreillette gauche (p= 0,02) était significativement associée à l’existence de troubles du rythme. Notre travail a montré l’intérêt du Holter ECG notamment chez les hypertendus. Il s’agit d’un examen complémentaire utile chez l’hypertendu pour détecter des troubles du rythme paroxystiques [15]. Il a permis de retrouver, dans notre travail, des troubles du rythme potentiellement graves avec une incidence thérapeutique et pronostique. Il y avait six cas de fibrillation atriale paroxystiques non présents sur l’ECG de repos. L’HTA et l’âge sont les principaux facteurs de risque de fibrillation atriale. La présence de fibrillation en soi augmente le risque d’AVC et, par ce biais, conduit à une brusque augmentation du niveau de risque cardio-vasculaire [14]. Il s’agissait aussi d’arythmies ventriculaires graves (tachycardie ventriculaire) signifiant le risque accru de mort subite retrouvé au cours de l´HTA [15]. L’intérêt du Holter ECG réside aussi dans l´étude de la variabilité de la fréquence cardiaque qui est altérée au cours de l´HTA, traduisant une anomalie du système nerveux autonome [15] et dans la recherche d’une aggravation d’un trouble de la conduction préexistant. Les troubles de la conduction étaient rapportés dans un quart de notre population (25,96%). Parmi ces derniers, nous avions observé une prédominance de l’hémibloc antérieur et du bloc de branche droit bien que cela puisse être simplement lié à l’âge. Le retentissement de l’HTA sur l’oreillette gauche est un élément important de la cardiopathie hypertensive. Dans notre travail, la dilatation de l’oreillette gauche était retrouvée dans un tiers de notre population totale. Son apparition paraissait significativement associée à l’âge (p= 0,01).

## Conclusion

L’hypertension artérielle est une affection dont l’incidence est importante dans la population âgée. Les aspects électrocardiographiques et échocardiographiques sont caractérisés par l’hypertrophie ventriculaire, la fréquence des arythmies sur l’ECG de base mais surtout à l’enregistrement électrocardiographique de longue durée et l’hypertrophie ventriculaire gauche à l’échocardiographie.

### Etat des connaissances actuelles sur le sujet

- Nous savons déjà que l’hypertension artérielle est un des principaux facteurs de risque cardio-vasculaire au Sénégal. La plupart des études faites au Sénégal le classent en 2e ou 3e facteur en terme de prévalence après la dyslipidémie et la sédentarité;
- Le sujet âgé est sujet à plusieurs comorbidités et l’HTA est une cause principale de décès à cet âge de par les complications cardio-vasculaires surtout les accidents vasculaires cérébraux et l’insuffisance rénale.

### Contribution de notre étude à la connaissance

- L’intérêt du Holter ECG dans l’évaluation de ces patients hypertendus âgés, nous avons retrouvé plusieurs cas de fibrillation atriale paroxystique qui peuvent être un facteur de risque important de survenue d’accidents vasculaires cérébraux;
- Le niveau de contrôle de l’HTA chez ces patients âgé était très faible.
